# Nutritional, Physico-Chemical and Mechanical Characterization of Vegetable Fibers to Develop Fiber-Based Gel Foods

**DOI:** 10.3390/foods10051017

**Published:** 2021-05-07

**Authors:** Ana Teresa Noguerol, Marta Igual, M. Jesús Pagán-Moreno

**Affiliations:** Investigation and Innovation Group, Food Technology Department, Universitat Politècnica de València, Camino de Vera s/n, 46022 Valencia, Spain; annome@etsiamn.upv.es (A.T.N.); marigra@upvnet.upv.es (M.I.)

**Keywords:** vegetable fibers, minerals, antioxidant capacity, gelling agent, thickener

## Abstract

The aim of this research was to evaluate the nutritional and physico-chemical properties of six different vegetable fibers and explore the possibility of using them as a thickener or gelling agent in food. To determine the technological, nutritional and physical parameters, the following analyses were carried out: water-holding capacity, water retention capacity, swelling, fat absorption capacity, solubility, particle size, moisture, hygroscopicity, pH, water activity, bulk density, porosity, antioxidant activity, phenolic compounds and mineral content. Gels were prepared at concentrations from 4% to 7% at 5 °C and analyzed at 25 °C before and after treatment at 65 °C for 20 min. A back extrusion test, texture profile analysis and rheology were performed and the pH value, water content and color were analyzed. As a result, all the samples generally showed significant differences in all the tested parameters. Hydration properties were different in all the tested samples, but the high values found for chia flour and citrus fiber are highlighted in functional terms. Moreover, chia flour was a source of minerals with high Fe, Mn and Cu contents. In gels, significant differences were found in the textural and rheological properties among the samples, and also due to the heat treatment used (65 °C, 20 min). As a result, chia flour, citrus, potato and pea fibers showed more appropriate characteristics for thickening. Moreover, potato fiber at high concentrations and both combinations of fibers (pea, cane sugar and bamboo fiber and bamboo, psyllium and citric fiber) were more suitable for gelling agents to be used in food products.

## 1. Introduction

Consumer interest in health, sustainability aspects of their way of life and their diet is growing. They demand more natural, less processed food, which are made using ingredients that are not perceived as negative [1]. Moreover, E numbers or a long ingredients list with scientific names shown on labels does not appeal to consumers; this negatively affects consumers’ perception regarding their naturalness and their impacts on health and wellness [2]. Therefore, these ingredients should be replaced with others that consumers perceive as being healthier, such as fibers [3].

It is a well-known fact that hydrocolloids change the physical properties of solutions to form gels, or enable thickening, emulsification, coating and stabilization, and they also provide viscosity and play a role in developing food with high satiating capacity [4]. Furthermore, hydrocolloids are of great importance in gel network formation since they can act as carriers for bioactive food ingredients, which usually have low mobility and diffusion rates because they are trapped by gel networks [5]. Besides functional qualities, hydrocolloids have also received enthusiastic support due largely to their dietary fiber (DF) aspect [4]. In this sense, it is important for its beneficial effects on health, as well as prebiotic ingredients and their technological attributes, such as water binding, gelling and structure building. Therefore, they can be used as a low-calorie sweetener, fat replacer or texture modifier [4,6]. For this reason, the interest shown in plants high in DF and natural antioxidants has increased in recent years [7]. Moreover, eating them offers several health benefits, including body weight control, serum lipid and cholesterol reduction, controlled postprandial glucose responses and colorectal cancer prevention [8].

For these reasons, designing future food structures by structuring food colloids such as fiber offers high theoretic and application value to use them, for example, in functional food products for elderly people with swallowing problems, special food products for diabetic patients or artificial plant-based meats for vegan and vegetarians [9].

The main purpose of this research was to evaluate the nutritional and physicochemical properties of different vegetable fibers, and the possibility of using them as a thickener or gelling agent in food.

## 2. Materials and Methods

### 2.1. Raw Materials

The samples used in this study were six different vegetable sources of DFs supplied by the company Productos Pilarica S.A., Paterna, Spain. Table 1 depicts the name, ingredients and proximate compositions of the samples, as indicated by their producers.

### 2.2. Physico-Chemical Analysis

Moisture (x_w_) (g water/100 g sample) was determined by vacuum oven drying (Vaciotem, J.P. Selecta, Spain) at 70 °C until constant weight.

The water activity (a_w_) of samples was analyzed by AquaLab PRE LabFerrer equipment (Pullman, Washington, DC, USA).

Hygroscopicity (Hg) was determined according to Cai and Corke [10].

The pH of samples was analyzed on dispersions (10% *w*/*v*) in distilled water following Bender et al. [11].

Sample particle size distribution was determined according to Standard ISO13320 (AENOR 2009) with a particle size analyzer (Malvern Instruments Ltd., Mastersizer 2000, Worcestershire, UK) equipped with a dry sample dispersion unit (Malvern Instruments Ltd., Scirocco 2000). Particle size distribution was characterized by the volume mean diameter (D [4, 3]) and standard percentiles d (0.1), d (0.5) and d (0.9).

Porosity (ε) was determined from true (ρ) and bulk (ρ_b_) densities according to Agudelo et al. [12] with slight modifications. To determine bulk density (ρ_b_), about 2 g of powder were placed inside a 10 mL-graduated test tube and the occupied volume was noted. Bulk density was calculated by dividing the mass of powder by the occupied volume, and was expressed as g/L. The real density of samples was established by a helium pycnometer (AccPyc 1330, Micromeritics, Norcross, GA, USA).

### 2.3. Hydration Properties

Water-holding capacity (WHC) and water retention capacity (WRC) were described in line with by Raghavendra et al. [13] and Chantaro et al. [14], respectively.

Swelling water capacity (SWC) and fat adsorption capacity (FAC) were described according to Navarro-González et al. [15] with minor modifications. For SWC, 1 g of sample was placed inside a graduated test tube and was hydrated with 20 mL of distilled water. Samples were stored for 18 h at 25 °C after recording the bed volume. SWC was expressed as volume mL/g sample. For the FAC determination, 4 g of samples were placed inside a centrifuge tube with 24× *g* of sunflower oil. Contents were stirred for 30 sec every 5 min for 30 min. Later samples were centrifuged at 1600× *g* for 25 min. Free oil was decanted and FAC was expressed as g oil/g sample.

The water solubility index (WSI) was analyzed according to the method of Mahdavi et al. [16] with minor modifications. Approximately 1 g of sample was mixed in centrifuge tubes with 30 mL of distilled water for 5 min until mixtures were homogeneous. Solutions were then incubated at 37 °C in a water bath for 30 min. Then, tubes were centrifuged at 17,640× *g* for 20 min at 4 °C. Supernatants were collected and dried in an oven at 100 °C until constant weight was achieved. The results are expressed as a percentage.

### 2.4. Antioxidant Capacity and Phenolic Compounds

Antioxidant capacity (AC) was assessed using DPPH method following the methodology of Igual et al. [17]. UV-visible spectrophotometer (Thermo Electron Corporation, Waltham, MA, USA) was used for the absorbance at 515 nm. The final results were expressed as milligram trolox equivalents (TE) per 100 g (mg TE/100 g).

Total phenol content (PC) was carried out according to Agudelo et al. [12]. Absorbance was measured at 765 nm in a UV-visible spectrophotometer (Thermo Scientific, Helios Zeta UV-Vis, Loughborough, UK). The total phenolic content was expressed as mg of gallic acid equivalents (GAE) (Sigma-Aldrich, Steinheim, Germany) per 100 g of sample.

### 2.5. Ash and Mineral Analysis

The total ash content was determined following the method 930.05 of AOAC procedures [18]. In total, 500 mg of sample were incinerated at high pressure in a microwave oven (Muffle P Selecta Mod.367PE) for 24 h at 550 °C, and ash was gravimetrically quantified.

The multimineral determination was analyzed in an inductively coupled plasma optical emission spectrometer, model 700 Series ICP-OES of Agilent Technologies (Santa Clara, USA), equipped with axial viewing and a charge coupled device detector [19]. Mineral compositions (macro- and microelements) were expressed as mg/100 g.

### 2.6. Gel Preparation

Samples were dissolved in cold water (5 °C) for 30 min at concentrations of 4%, 5%, 6% and 7% and were then divided into two batches. One was directly stored for 24 h at 5 °C until gel stabilization. However, the other batch was heated at 65 °C for 20 min before being stored for 24 h at 5 °C. The samples were tempered until reaching 25 °C for their analyses.

### 2.7. Gel Analysis: Water Content, pH, Color, Texture and Rheology

Water content (x_w_) (g water/100 g sample) was determined as in the powder fibers.

The pH of the gel samples was measured by a pH-meter Crison MultiMeter MM 41 (Hach Lange, Barcelona, Spain).

In order to determine the color of gel translucency, CIE*L*a*b* colors were measured according to García-Segovia et al. [19]. The color differences (∆E) associated with heating were calculated for each sample.

Textural characteristics were evaluated by a TA-XT2 Texture Analyzer (Stable Micro Systems Ltd., Godalming, UK). The back extrusion test was performed following the method described by Cevoli et al. [20] with minor modifications. The employed extrusion disc had a diameter of 25.4 mm, and was positioned centrally over the sample’s plastic container (diameter: 50 mm, height: 75 mm) with 40 g of sample (approximately 30 mm high). The test was performed at a depth of 50% at the 1 mm/s test speed. The attributes calculated from the force-deformation curve were the area under the curve up or consistency (N s), the maximum force or firmness (N), the maximum negative force or cohesiveness (N) and the resistance to flow off the disc or viscosity (N s).

The flow behavior of gels was measured on samples with back extrusion consistency results lower than 100 N s by following the method described by Cevoli et al. [20] with slight modifications. Flow curves were performed using a Kinexus pro^+^ rotational rheometer (Malvern Instruments, Worcestershire, UK), equipped with a system of coaxial cylinders (C25/PC25), and the rSpace software. Then, 20 mL of sample was loaded into the geometry and rested for 3 min to achieve temperature equilibrium (25 °C) and stress relaxation in a heat-controlled sample stage (Peltier Cylinder Cartridge, Malvern Instruments, Worcestershire, UK). Samples were exposed to a logarithmically increase shear rate from 0 to 200 s^−1^ in 3 min. Finally, consistency (k), the flow behavior index (*n*) and the apparent viscosity at a 50 s^−1^ shear rate (*η*_ap_) were calculated [21]. The analyses of gels were performed in triplicate for each concentration sample.

In total 40 g of each concentration of the gel preparations was weighed on cylindrical plastic glass (diameter: 50 mm, height: 75 mm). Gel structures were removed from the container before analyzing and a texture profile analysis (TPA) was performed with a TA-XT2 Texture Analyzer (Stable Micro Systems Ltd., Godalming, UK) and the Texture Exponent software (version 6.1.12.0). A double compression cycle test was run up to 50% strain compression of the original portion height in an aluminum cylinder probe (diameter: 75 mm) following the method described by Ağar et al. [22]. This analysis was performed only on samples with back extrusion consistency results over 100 N s.

### 2.8. Statistical Analysis

An analysis of variance (ANOVA), with a 95% confidence level (*p* < 0.05), was applied to evaluate the differences among samples using the Statgraphics Centurion XVII software, version 17.2.04. Furthermore, a correlation analysis among parameters, with a 95% significance level, was achieved.

## 3. Results and Discussion

### 3.1. DF Analysis

Table 2 shows the physico-chemical properties of the DF samples. In general, significant (*p* < 0.05) differences were found among all the samples. FBPC and FPE showed the highest x_w_, and no significant (*p* < 0.05) differences. The sample with the significantly (*p* < 0.05) lowest x_w_ was FC. The FC value was lower than those reported by de Moraes Crizel [6] for orange fibers. All the DF samples presented significant (*p* < 0.05) differences in the a_w_ values, with FPE and FP displaying higher a_w_. This would mean that their shelf life would be shorter because microorganisms could increase during storage. On the contrary, except for FPE and FP, the samples’ a_w_ values fell within the ideal range (0.11 to 0.40) to avoid microorganism growth and degradation reactions [6]. Significant differences were also observed between the pH values (*p* < 0.05). FCH was the sample with the highest pH and FC had the lowest pH. The sample with the lowest Hg was FCH. This value was significantly lower than those for the other samples (*p* < 0.05). According to Moghbeli et al. [23], lower hygroscopicity could be more positive because of its importance for the flowability factor during storage.

The ρ_b_ and ε values are shown in Table 2. In general, significant differences appeared in these parameters (*p* < 0.05). The sample with the highest ρ_b_ was FBPC and, at the same time, it was the sample with the lowest ε, although no significant differences were observed in ε between FBPC and FCH (*p* > 0.05). The lowest ρ_b_ was for FP. This value was slightly lower than that obtained by Huber et al. [24] for potato fiber. Both FPESB and FPE had similar ρ_b_ values to potato fiber and lower values compared to pea fiber [24].

**Figure 1:**
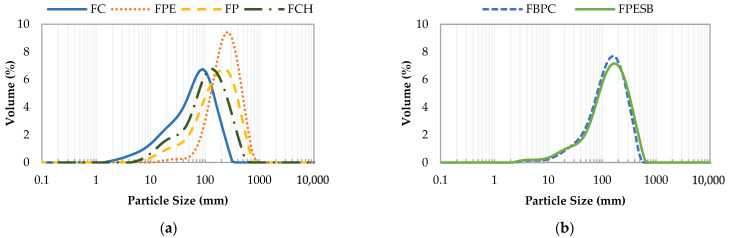
Volume of the particle size distributions (representative curves) of the studied fibers, where (**a**) presents the vegetable DF samples of one different source and (**b**) denotes the samples made with a combination of vegetable DFs.

Figure 1 shows the samples’ particle size distribution. Figure 1a indicates the vegetable DF samples of a different source. In this figure, the largest and the homogeneous particle size of samples are presented by FPE. The particles range lies between 60 and 830 μm, although there is a narrow particles range between 12 and 60 μm. This sample also has the highest volume mean diameter value (D [4, 3]) and standard percentiles d (0.1), d (0.5) and d (0.9) (Table 2). For FP, the particle size is distributed between 2.5 and 724 μm, although it concentrates the most between 72–724 μm. FCH shows a more concentrated particle size range between 40 and 550 μm, but similar to FP, its particles range is narrower, between 5–40 μm. The more marked peak appears for FC, which means that the particle size is more heterogeneous. We can observe that the more concentrated range in FC lies between 40 and 363 μm, but it presents the largest volume of a small particles size, between 1.9 and 40 μm. For both vegetable DF combinations (Figure 1b), the particle size distribution is similar, between 2.9 and 550 μm, but the FPESB sample has a higher D [4, 3] (Table 2). Finally, in Figure 1a,b, all the samples vastly differ. This is also observed in Table 2, where significant (*p* < 0.05) differences in D [4, 3], d (0.1), d (0.5) and d (0.9) are shown for all the samples. According to Rosell et al. [25], it is vital to know the particle size distribution of DFs because this parameter can determine fiber functionality and its role in the digestive tract (transit time, fermentation, fecal excretion).

**Figure 2:**
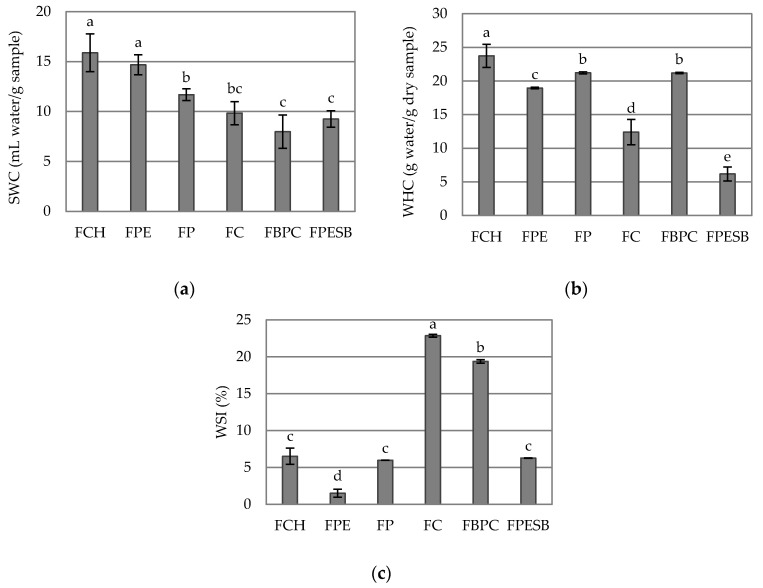
Mean values and standard deviations of (**a**) the swelling capacity (SWC), (**b**) the water-holding capacity (WHC) and (**c**) the solubility (WSI) of the DF samples. Letters indicate the homogeneous groups established by the ANOVA (*p* < 0.05).

Analyzing the hydration properties is important because they can determine the fate of DF in the digestive tract and explain some of its physiological effects [26]. Figure 2 shows the hydration properties (a) SWC, (b) WHC and (c) WSI of the tested DF samples. SWC is the volume occupied by a known weight of fiber under the applied condition. The SWC values of FCH and FPE were significantly higher than for the other samples (*p* < 0.05) (Figure 2a). The SWC of FPE was comparable to that shown by Huber et al. [24] for pea fiber, although the FP results were higher than potato fiber SWC reported by this author, but similar to that indicated by Kaack et al. [27] for potato pulp fiber. Wang et al. [28] obtained higher results than FC SWC for different sources of citrus fiber.

Rosell et al. [25] indicated that WHC was the amount of water retained by samples without being subjected to any stress. All the samples showed significant differences for the water-holding capacity (*p* < 0.05) (Figure 2b), except between FP and FBPC, and the highest WHC was shown by FCH, while FPESB presented the lowest value. Mancebo et al. [29] reported lower WHC for pea and potato fibers than the results herein shown for FPE and FP. However, the results reported by Wang et al. [28] for different citrus fibers were higher than the FC WHC result.

For WSI (Figure 2c), FC showed higher solubility, with significant (*p* < 0.05) differences among the other samples. The differences in the WSI of samples could be related to the nature of the glycidyl component and the structural characteristics of fiber [6]. Moreover, high solubility can inhibit the digestion and absorption of nutrients (glucose and cholesterol) from the gut [26,30]. The results of WSI for FC was lower than the results reported by de Moraes Crizel et al. [6] for DF from orange.

**Figure 3:**
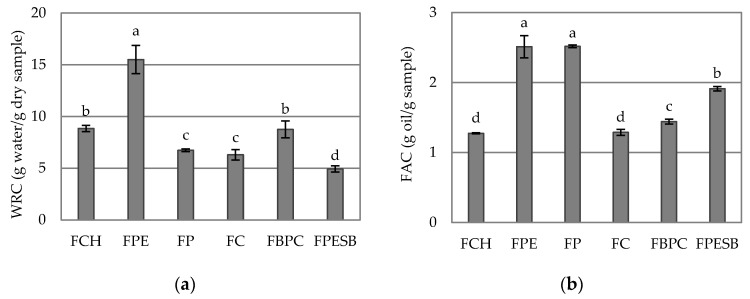
Mean values and standard deviations of (**a**) water retention capacity (WRC) and (**b**) fat absorption capacity (FAC) of DF samples. Letters indicate homogeneous groups established by the ANOVA (*p* < 0.05).

The ability of DF to retain water when subjected to an external force such as centrifugation is presented by WRC [8]. Both SWC and WRC can provide an overview of fiber hydration, and it is vital to know if fiber is suitable to be used in supplemented foods [26]. Figure 3a depicts the WRC values of studied samples, where we can see that the DF whose WRC was significantly (*p* < 0.05) higher was FPE, and the significant (*p* < 0.05) lowest WRC was for FPESB. These results are notably lower than those reported by Lan et al. [31] for the DF isolated from *P. odoratum* by drying in the sun. However, the FPE and FP values were higher than those obtained for potato and pea fibers according to Huber et al. [24]. WRC was lower for FCH than the 15.41 g water/g sample of chia fiber [32]. Moreover, a DF with high WRC can be used as a functional food ingredient to cut calories, avoid syneresis and modify both the viscosity and texture of processed food [6,33].

The ability of the fiber to absorb fat or oil lies in FAC. Figure 3b shows the FAC values of the studied samples. The samples with the highest FAC were FPE and FP, and no significant differences were found between them (*p* > 0.05). These results were similar to those shown by Huber et al. [24] for pea and potato fiber, and also to the pea fiber FAC reported by Mancebo et al. [29]. These values were notably higher than those reported by Navarro-González et al. [15] in tomato peel fiber. However, FCH and FC had the lowest FAC values, and no significant differences appeared between them (*p* > 0.05); however, the results reported by Wang et al. [28] for citrus fibers were higher. For FCH, higher results were found for chia fiber (2.02 g oil/g sample) [32]. It is vital to know this parameter because it means that high FAC could prevent fat loss during cooking, and also in nutrition by absorbing fat in the intestinal lumen which lowers cholesterol, and can also help to retain food flavor [6,15,30]. Therefore, the DF samples, which generally presented better hydration properties, were FPE, FP, FCH and FBPC for water binding, and FP and FPE for oil retention.

WSI presented a significant negative Pearson’s correlation for particle size (D [4, 3], d (0.1), d (0.5) and d (0.9)) (*p* < 0.05), and the higher WSI was, the smaller the particle size became: −0.7902, −0.6083, −0.7576 and −0.8342, respectively. However, FAC and WRC presented a significant (*p* < 0.05) positive correlation with particle size (D [4, 3], d (0.1), d (0.5), d (0.9)): 0.8636, 0.7059, 0.8481 and 0.8873 for FAC and 0.6866, 0.8658, 0.7210 and 0.5847 for WRC, respectively. This means that when the samples’ particle size grew, the absorption capacity of oil and water increased. Authors such as Lan et al. [31] indicated that an increased particle size is related to a better ability to bind oil and water, although this effect cannot be generalized because the hydration properties of DF were related to the chemical structure of component polysaccharides, and to other factors such as porosity, particle size, ionic forms, pH, temperature, ionic strength, type of ions in solution and stresses on fibers [34]. Furthermore, FAC showed positive correlations with ε (0.8219) when ε presents high values, and the capacity of DF to absorb oil increased. Finally, Hg showed negative correlations with hydration properties (WHC and SWC), −0.7071 and −0.8322, respectively, and higher Hg values implied less ability to absorb water. These results showed how variable fibers are and the importance of analyzing the characteristic of each type of fiber, as their composition plays a key role [14,25,35].

**Figure 4:**
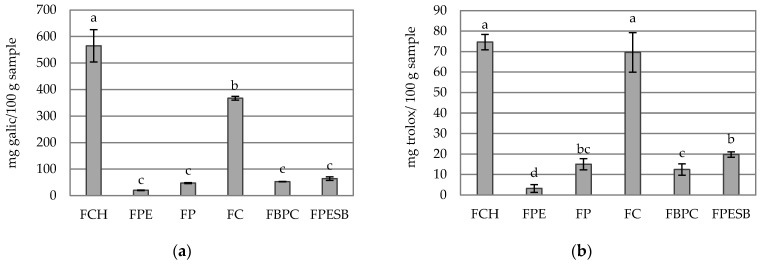
Mean values and standard deviations of (**a**) the samples’ total phenol content and (**b**) antioxidant capacity. Letters indicate the homogeneous groups established by the ANOVA (*p* < 0.05).

Figure 4 shows the total phenol content (a) and the antioxidant capacity (b) of the tested samples. The higher values of the total phenol content and antioxidant capacity in the FCH and FC samples are quite remarkable. FCH has the highest phenol content with significant differences compared to the other samples (Figure 3a). However, these differences are not significant compared to FC in antioxidant terms (Figure 3b). The FC total phenol content is similar to the total phenol concentrations in grapefruit powder [12] and for orange freeze-drying [36]. The antioxidant capacity values of FP fall within the same range as other studies performed with soluble dietary potato fiber [37]. Pearson’s correlation between total phenol content and antioxidant capacity was 0.9538. Thus, the total phenol content has a strong effect on the antioxidant capacity, as observed in other studies with Lulo (*Solanum quitoense*) [38] and grapefruit [39]. FCH and FC can be an important functional fiber if employed as a thickener or gelling agent because they have a high total phenol content and a good antioxidant capacity.

Table 3 shows the ash content, total minerals and mineral contents of the DF samples. All the samples generally showed a high mineral content. Both ash content and total mineral content were significantly different in all the studied samples (*p* < 0.05), and the FCH sample presented the highest content for both ash and minerals. It could be related to FCH, which is not an isolated dietary fiber, because it contains chia seed flour, which has a high percentage of total dietary fiber (Table 1). The K value in FP, FBPC and FPESB, whose formulation includes sugar cane, was similar to some results obtained by Chong et al. [40] for sugar cane fiber, and by Ma and Mu [8] for DFs, obtained from de-oiled cumin. FPE, FP and FPESB have obtained comparable values for Zn [40], as have FPE and FBPC for Ca [8]. Llorent-Martínez et al. [41] reported similar chia seed values to those observed for FCH for minerals P, Mg and Zn, but the FCH values for Ca, Cu and Na were higher, and the Na content was much higher in FCH. However, the Fe, Mn and K contents of FCH were lower [41]. Some values should be highlighted, such as Fe content, and also other trace elements, such as Mn, Zn and Cu for FCH, because Fe and Zn deficiency is a worldwide health problem, with about 30–33% of the world’s population at risk, particularly in underdeveloped countries [42].

SWC presented a significant Pearson’s correlation when related to P, Na, Zn and Cu (*p* < 0.05), and the higher SWC, the higher the content of these minerals: 0.6257, 0.8272, 0.6617 and 0.6332, respectively. WRC also correlated positively with Na (0.8153) (*p* < 0.05). However, negative correlations were found between WHC and Se (−0.6206), and between WSI and Na (−0.6830), and also when FAC was related to K and Ca (−0.6885 and −0.6896, respectively). Antioxidant capacity also showed positive correlations when associated with ash, total mineral content, P, K, Ca, Mg, Cu and Mn (0.7928, 0.8770, 0.6995, 0.8211, 0.9449 and 0.6749, respectively) (*p* < 0.05). At the same time, total phenol content also presented positive correlations with ash, total mineral content, P, K, Ca, Mg, Zn, Cu and Mn (0.8895, 0.9561, 0.8464, 0. 9217, 0.8621, 0.8101, 0.7792, 0.8196 and 0.7591, respectively) (*p* < 0.05). Although soluble and insoluble DF, and components such as polyphenols, are associated with a lower mineral absorption in the small intestine because of binding and/or physical entrapment, this is believed to be compensated by the fermentation of fibers in the colon by gut microflora, as short-chain fatty acids are produced that can release trapped minerals to increase the absorptive surface area and, hence, improve their absorption. This situation has been observed to be significant if deficient [43].

### 3.2. Gel Analysis

Gels were prepared from each fiber sample at concentrations of 4%, 5%, 6% and 7% to know the physico-chemical and mechanical properties and their possible use in food products. Table 4 depicts the physico-chemical properties (x_w_ and pH) and the gel samples’ color at each concentration. Samples’ water content (x_w_) showed a decrease in both (with and without heat treatment) when fiber concentration rose. Moreover, an interaction took place between the fiber concentration and the DF samples. For fiber sample FPE, the decrease was less intense, and this fiber presented the highest x_w_ at all the tested concentrations both with and without heat treatment, with significantly higher values at concentrations of 5%, 6% and 7% than the other fiber samples when gels were heated (*p* < 0.05). This could be because the FPE fiber presented the lowest solubility (Figure 2c), and thus, left more free water. In general terms, no significant effect on the x_w_ of the gel concentrations tested per fiber was observed due to heat treatment (*p* > 0.05).

Samples’ pH value was significantly different in both cases with and without heat treatment (*p* < 0.05) (Table 4), and the pH range went from 6.56 to 4.14. Only for dispersion, no significant differences were found (*p* > 0.05) for samples 4, 5 and 6% made with FPE and FPESB. For 7% gel, the pH of the FPESB sample was higher pH than the FPE gel (*p* < 0.05). In both cases, the different dispersions made with the FCH sample presented the highest pH, and the lowest pH was observed in the FC fiber dispersions, which agrees with the pH values observed in the 10% *w*/*v* dispersion of fibers (Table 2). For pH, an interaction between the concentration and fiber sample also took place, and the pH values were slightly lowered for all the fiber samples (*p* < 0.05) when concentrations rose. As in x_w_, no significant effect on pH was generally observed on the dispersion concentrations for each fiber due to heat treatment, except for FCH, which had a slightly higher pH in the heated sample dispersions (*p* < 0.05).

Generally speaking, in the samples with lower fiber content, solid content was precipitated, but the uniformity of suspensions markedly increased when concentration rose. This could be due to the fact that samples are mixtures of whole cells and dispersed cell wall materials, and it has been reported that these materials usually form non homogeneous suspensions, unlike smashed cellular material that forms homogeneous fibrous networks [44]. However, homogeneity increased while fiber concentration increased due to the mixture’s saturation.

**Figure 5:**
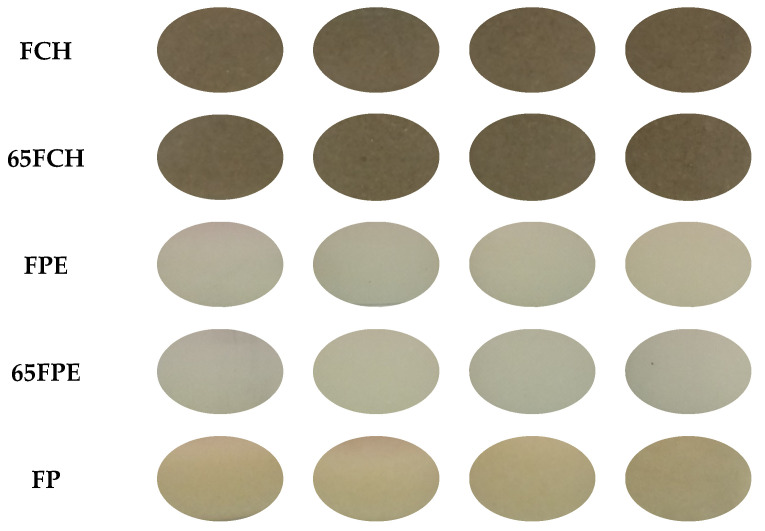

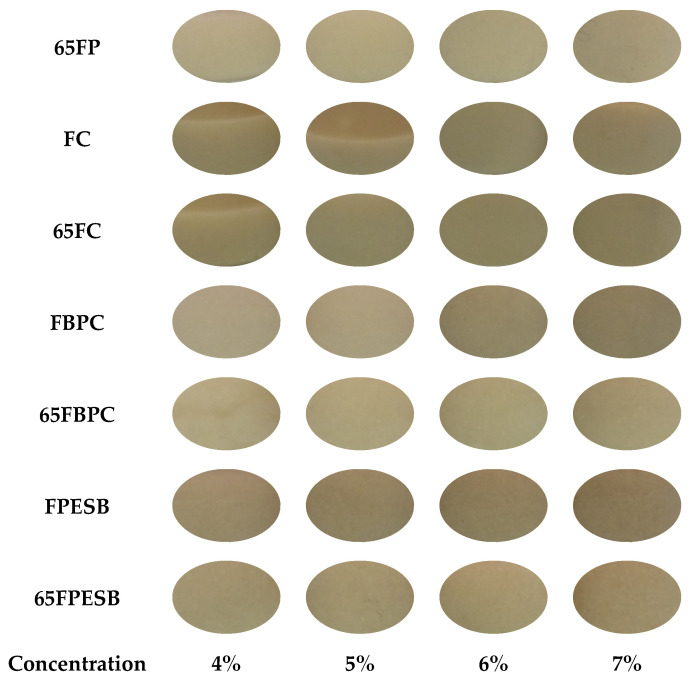
Color gel images at the tested concentrations.

The values of the colorimetric parameters (L*, a* and b*) of the formulated gels are presented in Table 4. To illustrate the color of suspensions, the images of each formulated sample are shown in Figure 5, both with and without heat treatment. We can see that samples totally differed, but parameters L*, a* and b* significantly increased with a higher fiber concentration for all the samples in both cases, with and without heat treatment (*p* < 0.05). It has been reported that color parameters increase in homogenized suspensions and are more saturated in red and yellow [44], like the results herein shown. Moreover, an increase in the color parameters was also related to the employed fiber, and the FCH suspensions were those with higher a* values in the untreated and heated samples (*p* < 0.05). Yellowness (b*) was the most different value in the samples (*p* < 0.05) in both the heated and unheated samples, except for 6% and 7% of the FPE and FBPC unheated samples and for 7% of the FC and FCH heated samples (*p* > 0.05). Figure 5 generally shows no differences between the heated or unheated samples. However, color differences (∆*E* > 3) appeared for samples at 4% and 5% of FBPC, and at 5%, 6% and 7% of FPE (Table 4), which means that these differences are visibly perceptible [45]. Furthermore, Pearson’s correlations were observed between the color parameters of both suspensions (heated and unheated) and mineral content. An increase in L* is related to a higher Se content of (0.7163), while an increase in a* (redness) is related to higher P, K, Mg, Zn, Fe, Cu and Mn contents and to total content of minerals (0.9419, 0.9036, 0.9635, 0.9290, 0.8084, 0.9373 and 0.8986, respectively). For b*, a positively correlation was also found with P, K, Ca, Fe and Cu (0.6684, 0.7529, 0.8431, 0.6292, 0.6245 and 0.8242, respectively).

The back extrusion assay was performed as in a previous study to elucidate the mechanical properties of fiber suspensions. This assay showed that not only did the consistency and firmness of gels increase as the concentration of DFs rose, but also the viscosity and cohesiveness in all the samples (Table 5). For all the samples and texture parameters, an interaction occurred between concentration and temperature, which were also related to the used fiber. A significant (*p* < 0.05) increase in all the parameters from the concentration of 4% was observed, except for all the parameters of the heated and unheated suspensions, where FC remained stable at all the concentrations. This increase was significantly greater when suspensions were formulated with the FBPC and FPESB fibers for all the parameters. In this way, the sample which presented the greatest consistency was 65FBPC (7%). The consistency, firmness and cohesiveness values were higher in 65FBPC (4%), but this sample had lower viscosity than that found by Cevoli et al. [20] for xanthan gum (4%). When comparing it to 65FPESB (4%), both showed similar consistency and cohesiveness, and 65FPESB (4%) obtained a higher strength and lower viscosity values. The sample with the lowest consistency and firmness was 4% FC for both heated and unheated conditions. Angioloni and Collar [46] reported a similar result to FC and 65FC (4% and 5%) in a pectin liquid-like gel prepared at 5 °C and 95 °C, and also similar results to 4% FP and FPE in a liquid-like gel prepared with pectin and FOS or GOS. Furthermore, the viscosity index and cohesiveness of these samples were also comparable to those found by these authors [46]. Moreover, the importance of determining consistency and viscosity is because they are related to the coverage in mouth of gelled products, as indicated by Igual et al. [47] and Igual et al. [48] for jam.

In order to continue characterizing samples after obtaining the back extrusion assay results, the samples with a lower consistency than 100 N s were rheologically characterized by the flow curve analysis. The adjustment of shear stress and the shear rate of all the data were fitted to Ostwald’s power law, where *k* and *n* indicate consistency and flow behavior, respectively. In addition, *R*^2^ (goodness of fit) values over 0.9 were obtained (Table 6). The change in *k* showed that increased consistency was related to a higher fiber concentration for all the samples, as Su et al. [49] indicated for citrus fiber-oil dispersions. At the same time, consistency increased when samples were heated, as shown by the back extrusion results, except for the samples prepared with FCH (Table 5). The gels formulated with FPBC and FPESB presented higher consistency. Therefore, both these fibers and those with a *k* over 20 Pa are identified with semisolid or spoonable foods [50], and have gelling properties for their possible use in different food products, such as fat replacers and/or to modify the texture of meat analogs, among others. However, the other studied fibers (*k* values between 0.21 and 3.6) are associated with liquids or drinkable food, and would be more suitably used as thickeners in soups, sauces and dressings. FC was the fiber with the lowest thickening power and FP had the highest (*p* < 0.05) at the highest tested concentration (7%). *n* was lower than 1, which means that samples behaved as pseudoplastic fluids. In general, no changes were observed in *n* due to increased fiber content, except for the unheated FC, FPE and FP samples, and the heated FC and FPE samples, for which a decrease occurred with increased fiber. This parameter depended on particle size distribution [44]. For this reason, negative Pearson’s correlations were observed between *n* and particle size parameters (D [4, 3], d (0.1) and (0.5); −0.6968, −0.6934 and −0.7345, respectively), which means that diminishing shear thinning behavior (higher *n* value) is related to reduced particle size. The result for the consistency of 65FPESB 7% was comparable to that reported by Santo-Domingo et al. [51] for artichoke dietary fiber, but shear thinning behavior (lower *n* value) was higher in artichoke fiber. In this study, the sample which displayed high consistency and good shear thinning behavior was 65FP 7%.

Table 6 offers the shear viscosity recorded at the shear rate (50 s^−1^) in accordance with the dispersion concentrations. According to Agarwal et al. [52], shear viscosity depends on the source, processing and microstructure of fibers. This table reveals that shear viscosity is also dependent on treatments, and the heated samples tended to be higher (*p* < 0.05), except for the FCH sample. At the highest studied concentration (7%), samples FBPC and FPESB displayed higher viscosity, and were higher when gels were heated. These viscosity results obtained with the different gel fibers are required to know in which model systems these fibers can be used as thickeners or gelling agents in order to investigate whether the inherent properties of the materials can impact the sensory characteristics of the texture analysis and taste perception.

**Figure 6:**
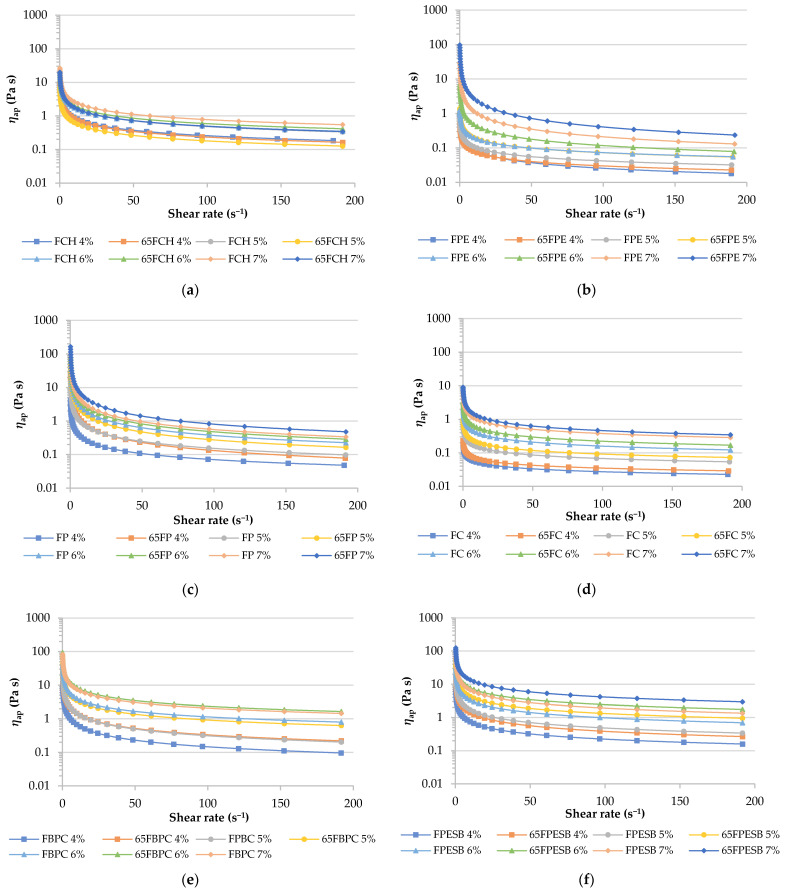
Apparent viscosity (*η*_ap_) vs. the shear rate of samples, where (**a**) shows the FCH, (**b**) FPE, (**c**) FP, (**d**) FC, (**e**) FBPC and (**f**) FPESB sample gels.

The effect of both concentration and temperature treatment on the *η*_ap_ trend is seen in Figure 6. Wang et al. [44] indicated that apparent viscosity increased with a rising fiber concentration, which also happened in the results herein shown. Moreover, the apparent viscosity trend increased in the heated samples for all the samples, except for those formulated with FCH and FC, which seemed to follow the same trend in both heated and unheated concentration samples (Figure 6a,d).

Pearson correlations were observed between hydration properties of gels (WHC, WRC, SWC, FAC and SWI) and mechanical properties (back extrusion and rheological properties) depending on how they have been formed (with or without heat treatment). When gels were formed without heating, SWC was negatively related to *η*_ap_ and *k* (−0.6688 and −0.6383, respectively). Moreover, FAC and WRC were also negatively related to *n* (−0.8853 and −0.6038, respectively); however, SWI showed positive correlation with *n* (0.6881). On the other hand, for heated gels, Pearson correlations were presented between WHC and back extrusion parameters, negative correlation with consistency (−0.6381) and firmness (−0.6011) and positive correlation with viscosity (0.6901) and cohesiveness (0.6194). Furthermore, an increase in the WHC was associated with less *η*_ap_ (−0.7472). Additionally, an increase in the *n* value was related to less FAC but high WSI (−0.8843 and 0.7713, respectively). Hence, the hydration properties of DFs are associated with gels structure; however, the type of DF and the treatment used to form the gel play an important role.

In order to finish characterization, the sample with consistency higher than 100 N s in the back extrusion analysis (65FBPC 7%) was analyzed by a texture profile analysis (TPA) because it presented a stable structure gel at this concentration. A resilience value of 0.17 ± 0.02 and a chewiness value of 1.5 ± 0.2 N were provided by the TPA analysis. Gel adhesiveness was negative (−1.6 ± 0.2 N s), which indicates that it forms a sticky gel. This result is comparable to those reported by Sharma et al. [53] for carrot puréed using modified corn starch and ThickenUp^®^. Adhesiveness is defined as a gel’s ability to adhere to a probe when it is withdrawn after the first compression. Therefore, this should be taken into account when making products because this texture might prove unpleasant for consumers because it adheres to palates, which requires making more effort to separate it [54]. Similar results for springiness (0.9105 ± 0.0119 mm) and cohesiveness (0.9105 ± 0.0119) have also been reported by Sharma et al. [53], but gel hardness (2.0 ± 0.4 N) was similar to the potato starch gels (PS54) reported by Torres et al. [55]. As a result, this fiber can be used as a gelling agent and to also adapt the texture of different foods due to its water-holding and water retention capacities, and also to its ability to form stable gels.

## 4. Conclusions

The results of this study revealed that all the DF samples had different physicochemical, hydrational, nutritional and mechanical properties. This is of great importance for the future use of this type of DF for the development of new food products such as meat analogue products, either to replace ingredients such as fat or to achieve new textures or improve/modify existing ones. In general, FPE and FCH showed good water-binding capacities and FP displayed good oil retention. We highlight the functional level values obtained for the FCH and FC samples. Moreover, in nutritional terms, FCH was a source of important minerals such as Fe, Mn and Cu. However, in suspension or gels, significant differences among samples in textural and rheological properties were found due to the employed treatment; as a result, FCH, FPE, FP and FC were more appropriate for thickening and FP at a high concentration, and both combinations of fiber (FBPC and FPESB) were more suitable to be used as gelling agents given their ability to form stable gels. In addition, the capacity of these DFs to change the texture without high temperatures can be highlighted. This enables them to modify the texture of different foods and to provide benefits of consuming DF.

## Figures and Tables

**Table 1:** Sample names, ingredients and proximate compositions expressed as g/100 g sample.

Sample	Name	Ingredients	Proteins	Lipids	Carbohydrates	TDF	IDF	SDF
FCH	Chia Flour	*Salvia hispanica* Seed Powder	30.95	12	49.04	45.5	43.1	3.5
FPE	Pea Fiber	Yellow pea fiber	5.19	0.3	93.41	43.8	36.7	7.1
FP	Potato Fiber	Synergetic combination of potato pure fiber	2.17	3.1	92.37	>60.0	>60.0	<1.0
FC	Citrus Fiber	Citrus Fiber	5.89	1.02	81.34	71.1	37.6	33.3
FBPC	Bamboo, Psyllium and Citric Fiber	Combination of bamboo, psyllium and citric fibers	0.613	0.5	98.287	45.6	42.1	3.5
FPESB	Pea, cane Sugar and Bamboo Fiber	Vegetable fibers: pea, cane sugar and bamboo	4.7	0.9	92.11	>60.0	>60.0	<1.0

TDF (total dietary fiber); IDF (insoluble dietary fiber); SDF (soluble dietary fiber).

**Table 2:** Mean values ± standard deviation of moisture (x_w_) (g_w_/100 g sample), water activity (a_w_), pH (dispersions 10% *w*/*v*), hygroscopicity (Hg) (g water/100 g dry solid), bulk density (ρ_b_) (g/L), porosity (ε), volume mean diameter D [4, 3] (μm) and the standard percentiles d (0.1), d (0.5) and d (0.9) of the studied samples.

	Vegetable Fiber Samples					
	FCH	FPE	FP	FC	FBPC	FPESB
x_w_	3.47 ± 0.06 ^d^	6.62 ± 0.03 ^a^	6.13 ± 0.12 ^b^	2.686 ± 0.118 ^e^	6.676 ± 0.104 ^a^	5.7 ± 0.3 ^c^
a_w_	0.372 ± 0.003 ^c^	0.5200 ± 0.0012 ^a^	0.4857 ± 0.0012 ^b^	0.1230 ± 0.0012 ^f^	0.3590 ± 0.0012 ^d^	0.342 ± 0.002 ^e^
pH	6.063 ± 0.015 ^a^	5.25 ± 0.06 ^d^	4.65 ± 0.04 ^e^	4.06 ± 0.02 ^f^	5.45 ± 0.02 ^c^	5.6633 ± 0.0115 ^b^
Hg	13.9 ± 0.3 ^d^	19.1 ± 0.6 ^c^	19.2 ± 0.8 ^c^	29.3 ± 0.3 ^a^	26.7 ± 0.7 ^b^	27.3 ± 0.2 ^b^
ρ_b_	424 ± 30 ^b^	348 ± 19 ^c^	211 ± 8 ^d^	410 ± 27 ^b^	489 ± 17 ^a^	354 ± 10 ^c^
ε	70.7 ± 1.3 ^d^	76.9 ± 0.5 ^b^	86.63 ± 0.16 ^a^	73.22 ± 0.15 ^c^	69.22 ± 0.95 ^d^	77.51 ± 0.12 ^b^
D [4, 3]	126.9 ± 1.3 ^e^	249 ± 14 ^a^	184 ± 4 ^b^	71.8 ± 0.6 ^f^	142.6 ± 0.3 ^d^	156 ± 2 ^c^
d (0.1)	23.0 ± 0.3 ^d^	99 ± 3 ^a^	36 ± 4 ^b^	11.92 ± 0.07 ^e^	36.1 ± 0.3 ^b^	33.0 ± 0.4 ^c^
d (0.5)	104.3 ± 0.7 ^e^	224 ± 12 ^a^	155 ± 3 ^b^	60.2 ± 0.4 ^f^	124.4 ± 0.3 ^d^	132.9 ± 1.3 ^c^
d (0.9)	263 ± 3 ^d^	438 ± 29 ^a^	375 ± 6 ^b^	150 ± 2 ^e^	276.2 ± 0.9 ^d^	313 ± 6 ^c^

A different letter in the same row denotes a significant difference as determined by the LSD test (*p* < 0.05).

**Table 3:** Mean values ± standard deviations of ash (%), total minerals (mg/100 g sample) and mineral content (mg/100 g sample).

	Vegetable Fiber Samples					
	FCH	FPE	FP	FC	FBPC	FPESB
Ash (%)	8.01 ± 0.04 ^a^	1.1 ± 0.2 ^d^	2.36 ± 0.03 ^c^	2.9 ± 0.5 ^b^	0.60 ± 0.03 ^e^	2.29 ± 0.04 ^c^
Total minerals	2460 ± 22 ^a^	453 ± 79 ^d^	510 ± 42 ^d^	1227 ± 34 ^b^	316 ± 18 ^e^	746 ± 29 ^c^
P	582 ± 33 ^a^	29 ± 10 ^c^	26 ± 2 ^c^	60 ± 5 ^b^	7.9 ± 0.7 ^c^	32.9 ± 1.4 ^c^
K	639 ± 6 ^a^	42 ± 8 ^f^	101 ± 10 ^e^	241 ± 16 ^b^	167 ± 14 ^c^	141 ± 7 ^d^
Ca	672 ± 25 ^b^	71 ± 24 ^e^	181 ± 13 ^d^	784 ± 26 ^a^	96 ± 3 ^e^	340 ± 16 ^c^
Na	170 ± 12 ^b^	275 ± 39 ^a^	116 ± 12 ^c^	73 ± 2 ^d^	40 ± 4 ^e^	60 ± 3 ^d,e^
Mg	380 ± 23 ^a^	33 ± 12 ^d^	81 ± 7 ^c^	66 ± 5 ^c^	3.706 ± 1.002 ^e^	107 ± 4 ^b^
Zn	6.4 ± 0.9 ^a^	0.6 ± 0.3 ^b^	0,22 ± 0.05 ^b^	- ^b^	- ^b^	0.58 ± 0.03 ^b^
Fe	6.4 ± 0.6 ^a^	1.6 ± 0.9 ^c^	4.7 ± 0.3 ^b^	2.1 ± 0.2 ^c^	1.6 ± 0.3 ^c^	4.2 ± 0.5 ^b^
Mn	2.74 ± 0.14 ^a^	- ^c^	- ^c^	- ^c^	- ^c^	0.75 ± 0.06 ^b^
Cu	1.29 ± 0.07 ^a^	- ^b^	- ^b^	- ^b^	- ^b^	- ^b^
Se	- ^d^	0.046 ± 0.003 ^b^	0.0278 ± 0.0009 ^c^	0.058 ± 0.013 ^a^	- ^d^	- ^d^

A different letter in the same row is significantly different as determined by the LSD test (*p* < 0.05).

**Table 4:** Mean values ± standard deviations of the x_w_ (g/100 g sample), pH and color parameters (L*, a* and b*) of the formulated gels.

Sample	C	x_w_	pH	L*	a*	b*	∆E
FCH	4	96.35 ± 0.04 ^a,b,A^	6.44 ± 0.02 ^a,C,D^	26.0 ± 0.2 ^k,C^	2.51 ± 0.09 ^d,D^	9.5 ± 0.7 ^d,E^	
	5	95.48 ± 0.03 ^d,B^	6.38 ± 0.13 ^b,c,E,F^	27.3 ± 0.3 ^h,i,j,B^	3.07 ± 0.06 ^c,C^	11.2 ± 0.4 ^b,C,D^	
	6	95.37 ± 0.04 ^d,B^	6.41 ± 0.05 ^b,D,E^	29.4 ± 0.5 ^g,A^	3.58 ± 0.14 ^a,A^	12.0 ± 0.6 ^a,B,C^	
	7	93.52 ± 1.06 ^f,C^	6.4 ± 0.2 ^c,F^	29.0 ± 0.5 ^g,A^	3.19 ± 0.13 ^b,C^	11.12 ± 0.12 ^b,C,D^	
65FCH	4	96.21 ± 0.09 ^y,x,A^	6.56 ± 0.02 ^z,A^	26.6 ± 0.4 ^r,B,C^	2.66 ± 0.12 ^w,D^	10.7 ± 0.6 ^y,D^	1.4 (0.7) ^e,f,g,h^
	5	95.519 ± 0.116 ^v,u,B^	6.50 ± 0.19 ^y,B^	28.8 ± 1.5 ^s,A^	3.7 ± 0.4 ^z,A^	13.1100 ± 1.0012 ^z,A^	1.7 (0.9) ^d,e,f,g^
	6	94.94 ± 0.16 ^s,r,q,B^	6.452 ± 0.009 ^x,C^	29.2 ± 0.6 ^t,s,A^	3.51 ± 0.19 ^y,A,B^	12.8 ± 0.8 ^z,A,B^	1.3 (0.2) ^e,f,g,h,i^
	7	95.26 ± 0.06 ^u,t,s,B^	6.4 ± 0.3 ^x,C,D^	29.1 ± 0.5 ^t,s,A^	3.25 ± 0.19 ^x,B,C^	11.2 ± 0.9 ^y,C,D^	1.0 (0.4) ^f,g,h,i,j^
FPE	4	96.7 ± 0.3 ^a,A^	5.88 ± 0.03 ^g,A^	33.2 ± 0.6 ^e,D^	0.10 ± 0.03 ^l,D^	2.1 ± 0.3 ^m,C^	
	5	96.1 ± 0.3 ^b,c,B^	5.76 ± 0.13 ^h,B^	39.1 ± 0.3 ^c,B^	0.14 ± 0.03 ^l,D^	3.186 ± 0.113 ^j,k,A^	
	6	95.57 ± 0.07 ^d,C^	5.64 ± 0.06 ^i,C^	35.5 ± 1.9 ^d,C^	0.11 ± 0.04 ^l,D^	2.6 ± 0.5 ^l,B^	
	7	94.4 ± 0.5 ^e,D^	5.482 ± 0.108 ^j,D^	41.31 ± 1.05 ^a,b,A^	0.16 ± 0.05 ^l,C^	3.4 ± 0.2 ^j,A^	
65FPE	4	96.77 ± 0.13 ^z,A^	5.85 ± 0.04 ^s,A^	33.1 ± 1.7 ^v,D^	−0.060 ± 0.012 ^p,o,B,C^	0.65 ± 0.08 ^n,D^	2.1 (0.5) ^b,c,d,e^
	5	96.3845 ± 0.1103 ^z,y,A,B^	5.61 ± 0.09 ^p,C^	34.6 ± 0.7 ^w,v,C,D^	−0.052 ± 0.009 ^p,o,A,B^	0.74 ± 0.04 ^n,D^	5.5 (0.4) ^a^
	6	96.0 ± 0.4 ^x,w,B,C^	5.49 ± 0.06 ^o,D^	30.7± 1.3 ^u,E^	−0.08 ± 0.03 ^o,B^	0.63 ± 0.08 ^n,D^	6.5 (0.4) ^a^
	7	94.686 ± 0.112 ^q,p,o,D^	5.448 ± 0.118 ^n,D^	39 ± 2 ^y,x,B^	0.05 ± 0.03 ^q,p,o,A^	2.3 ± 0.4 ^p,B,C^	3.7 (0.7) ^b,c^
FP	4	96.3 ± 0.2 ^a,b,A^	5.04 ± 0.05 ^k,A^	28.12 ± 1.18 ^h,E^	0.514 ± 0.102 ^j,C^	4.4 ± 0.4 ^i,D^	
	5	95.39 ± 0.14 ^d,B^	4.944 ± 0.018 ^l,C^	33.9 ± 0.9 ^e,D^	0.912 ± 0.109 ^f,A,B^	7.6 ± 0.2 ^f,B^	
	6	94.9 ± 0.4 ^e,B,C^	4.91 ± 0.03 ^l,D^	38.35 ± 1.08 ^c,C^	0.87 ± 0.12 ^f,g,h,A,B^	7.9 ± 0.6 ^f,A,B^	
	7	94.6 ± 0.3 ^e,C^	4.9 ± 0.9 ^m,E^	41.6 ± 0.6 ^a,A^	1.08 ± 0.16 ^e,A^	8.7 ± 0.7 ^e,A^	
65FP	4	96.5 ± 0.8 ^z,y,A^	5.00 ± 0.03 ^m,B^	30.25 ± 1.06 ^u,t,E^	0.6 ± 0.3 ^t,s,C^	6.0 ± 0.9 ^t,C^	2.9 (0.5) ^b^
	5	95.36 ± 0.09 ^v,u,t,B^	4.930 ± 0.014 ^l,C,D^	33 ± 2 ^v,D^	0.5 ± 0.2 ^s,C^	6.1 ± 0.6 ^t,C^	1.6 (0.6) ^c,d,e,f^
	6	94.86 ± 0.15 ^r,q,p,B,C^	4.882 ± 0.013 ^k,E^	38 ± 2 ^x,C^	0.7 ± 0.2 ^u,t,s,B,C^	7.6 ± 0.9 ^v,u,B^	1.3 (0.9) ^e,f,g,h,i^
	7	94.2 ± 0.6 ^n,m,C^	4.9 ± 0.9 ^j,E^	39.5 ± 1.6 ^y,B^	0.7 ± 0.2 ^v,u,t,B,C^	8.1 ± 0.9 ^v,A,B^	2.8 (0.9) ^b^
FC	4	96.40 ± 0.06 ^a,b,A^	4.26 ± 0.02 ^n,A^	35.31 ± 0.13 ^d,G^	−0.12 ± 0.06 ^m,F^	7.49 ± 0.16 ^f,G^	
	5	95.52 ± 0.04 ^d,B^	4.184 ± 0.009 ^o,D^	38.85 ± 0.12 ^c,E^	0.32 ± 0.03 ^k,D^	9.62 ± 0.19 ^d,E^	
	6	94.50 ± 0.05 ^e,D^	4.180 ± 0.009 ^o,D^	40.5 ± 0.2 ^b,C^	0.41 ± 0.07 ^j,k,C^	10.7 ± 0.3 ^c,C^	
	7	93.80 ± 0.03 ^f,E^	4.14 ± 1.04 ^p,E^	42.1 ± 0.4 ^a,A^	0.79 ± 0.07 ^h,A^	12.09 ± 0.19 ^a,A^	
65FC	4	96.32 ± 0.17 ^y,x,A^	4.226 ± 0.015 ^i,B^	35.2 ± 0.3 ^w,G^	−0.06 ± 0.02 ^p,o,F^	7.20 ± 0.14 ^u,H^	0.43 (0.15) ^i,j^
	5	95.09 ± 0.06 ^t,s,r,C^	4.200 ± 0.002 ^i,h,C^	38.2 ± 0.4 ^y,x,F^	0.1940 ± 0.1108 ^r,q,E^	9.0380 ± 0.1114 ^w,F^	0.9 (0.4) ^g,h,i,j^
	6	94.50 ± 0.03 ^p,o,n,D^	4.180 ± 0.007 ^h,g,D^	39.4 ± 0.4 ^y,D^	0.54 ± 0.03 ^s,B^	9.9 ± 0.4 ^x,D^	1.4 (0.5) ^e,f,g,h^
	7	93.72 ± 0.03 ^l,E^	4.15 ± 1.05 ^g,E^	41.06 ± 0.13 ^z,B^	0.74 ± 0.06 ^v,u,t,A^	11.28 ± 0.09 ^y,B^	1.3 (0.4) ^e,f,g,h,i^
FBPC	4	96.50 ± 0.08 ^a,b,A^	6.09 ± 0.06 ^d,A^	17.0 ± 0.2 ^n,G^	−0.21 ± 0.03 ^n,D^	0.43 ± 0.08 ^n,G^	
	5	95.63 ± 0.03 ^c,d,B^	5.98 ± 0.02 ^e,C^	20.3 ± 0.4 ^m,F^	−0.118 ± 0.009 ^m,n,C^	0.77 ± 0.12 ^n,F^	
	6	94.68 ± 0.03 ^e,D^	5.99 ± 0.07 ^e,C^	26.7 ± 0.5 ^j,k,B^	0.10 ± 0.05 ^l,B^	2.78 ± 0.13 ^k,l,C^	
	7	93.72 ± 0.05 ^f,F^	5.9 ± 0.5 ^g,D^	28.0 ± 0.4 ^h,i,A^	0.19 ± 0.03 ^l,A^	3.43 ± 0.15 ^j,A^	
65FBPC	4	96.44 ± 0.06 ^z,y,A^	6.05 ± 0.05 ^w,B^	21.51 ± 1.18 ^o,E^	−0.11 ± 0.04 ^o,C^	1.6 ± 0.4 ^o,E^	5.6 (0.3) ^a^
	5	95.46 ± 0.09 ^v,u,t,C^	5.97 ± 0.02 ^v,C^	23.0 ± 0.7 ^p,D^	−0.08 ± 0.07 ^p,o,C^	2.2 ± 0.3 ^p,o,D^	3.1 (0.8) ^b^
	6	94.47 ± 0.05 ^o,n,E^	5.97 ± 0.06 ^v,C^	25.3 ± 0.2 ^q,C^	0.06 ± 0.02 ^q,p,o,B^	3.14 ± 0.18 ^q,B^	1.5 (0.6) ^d,e,f,g^
	7	93.78 ± 0.16 ^l,F^	5.9 ± 0.5 ^u,D^	28.1 ± 0.3 ^s,A^	0.1060 ± 0.0114 ^q,p,B^	3.53 ± 0.13 ^q,A^	0.4 (0.3) ^j^
FPESB	4	96.15 ± 0.06 ^b,A^	5.93 ± 0.02 ^f,A^	22.1 ± 0.8 ^l,E^	0.45 ± 0.04 ^j,E^	3.23 ± 0.08 ^j,D^	
	5	95.51 ± 0.09 ^d,C^	5.76 ± 0.02 ^h,C^	27.2 ± 0.4 ^i,j,D^	0.67 ± 0.07 ^i,C^	4.6 ± 0.3 ^h,i,C^	
	6	94.77 ± 0.13 ^e,D^	5.67 ± 0.02 ^i,E^	29.2 ± 0.5 ^g,C^	0.81 ± 0.09 ^g,h,B^	5.0 ± 0.3 ^g,h,B^	
	7	93.93 ± 0.06 ^f,E^	5.65 ± 0.02 ^i,E^	30.6 ± 0.2 ^f,B^	0.89 ± 0.03 ^f,g,A^	5.2 ± 0.3 ^g,B^	
65FPESB	4	96.23 ± 0.09 ^y,x,A^	5.93 ± 0.03 ^v,A^	20.2 ± 0.7 ^o,F^	0.33 ± 0.05 ^r,F^	2.5 ± 0.3 ^p,E^	2.5 (0.9) ^b,c,d^
	5	95.69 ± 0.07 ^w,v,B^	5.85 ± 0.04 ^u,t,B^	26.7 ± 0.4 ^r,D^	0.60 ± 0.03 ^t,s,D^	4.28 ± 0.07 ^r,C^	0.4 (0.2) ^i,j^
	6	94.63 ± 0.09 ^q,p,o,n,D^	5.77 ± 0.02 ^r,C^	29.1 ± 0.4 ^t,s,C^	0.81 ± 0.05 ^v,u,B^	5.0 ± 0.2 ^s,B^	0.71 (0.15) ^h,i,j^
	7	93.95 ± 0.16 ^m,l,E^	5.72 ± 0.02 ^q,D^	31.6 ± 0.5 ^u,A^	0.88 ± 0.06 ^v,A^	5.8 ± 0.2 ^t,A^	1.3 (0.3) ^e,f,g,h,i^

For the samples with the same conditions, the same lowercase letter in superscript in columns indicates the homogeneous groups established by ANOVA (*p* < 0.05) (a–n for unheated, z–o for heated samples). To compare the same sample with the temperature effect, the same capital superscript letter in columns indicates the homogeneous groups established by ANOVA (*p* < 0.05). ∆*E* indicates color differences associated with the heat treatment. C: Concentration (%).

**Table 5:** Mean values ± standard deviations of the back extrusion parameters of the formulated gels.

Sample	C	Consistency (N s)	Firmness (N)	Viscosity (N s)	Cohesiveness (N)
FCH	4	5.4 ± 0.5 ^h,i,j,C^	0.92 ± 0.04 ^h,i,E^	0.66 ± 0.05 ^f,g,D^	0.63 ± 0.06 ^g,h,i,D^
	5	6 ± 2 ^h,i,j,C^	1.0 ± 0.4 ^h,i,D,E^	0.74 ± 0.18 ^f,D^	0.80 ± 0.15 ^f,g,h,C,D^
	6	16 ± 3 ^e,f,A^	3.1 ± 0.7 ^d,A,B^	1.46 ± 0.17 ^d,A^	1.9 ± 0.3 ^e,A^
	7	16 ± 4 ^d,e,A^	3.3 ± 0.8 ^d,A^	1.4 ± 0.2 ^d,A^	1.8 ± 0.4 ^e,A,B^
65FCH	4	6.4 ± 0.9 ^q,p,o,n,C^	1.16 ± 0.17 ^o,n,D,E^	0.81 ± 0.05 ^s,r,C,D^	0.78 ± 0.15 ^q,p,C,D^
	5	10.1 ± 0.6 ^r,q,p,o,B^	1.74 ± 0.06 ^p,o,n,C,D^	0.98 ± 0.03 ^t,s,C^	1.09 ± 0.09 ^r,q,C^
	6	12.85 ± 1.19 ^s,r,q,A,B^	2.3 ± 0.3 ^q,p,o,B,C^	1.23 ± 0.09 ^u,t,B^	1.47 ± 0.09 ^s,r,B^
	7	15 ± 5 ^t,s,r,A^	3.1 ± 1.3 ^r,q,p,A,B^	1.3 ± 0.2 ^u,t,A,B^	1.6 ± 0.5 ^s,r,A,B^
FPE	4	1.04 ± 0.06 ^k,D^	0.20 ± 0.03 ^i,C^	0.065 ± 0.009 ^j,k,C^	0.146 ± 0.016 ^j,k,C^
	5	2.0 ± 0.4 ^j,k,D^	0.68 ± 0.19 ^h,i,C^	0.07 ± 0.02 ^k,C^	0.150 ± 0.015 ^j,k,C^
	6	5 ± 3 ^h,i,j,B,C,D^	2.0 ± 1.3 ^f,g,B,C^	0.15 ± 0.09 ^i,j,k,B,C^	0.3 ± 0.2 ^i,j,k,B,C^
	7	10 ± 3 ^g,B,C^	3.3 ± 0.7 ^d,A,B^	0.31 ± 0.06 ^h,i,j,B^	0.61 ± 0.06 ^g,h,i,B,C^
65FPE	4	1.6 ± 0. 3 ^o,n,D^	0.56 ± 0.14 ^o,n,C^	0.062 ± 0.003 ^p,C^	0.157 ± 0.009 ^o,C^
	5	4.1 ± 0.9 ^p,o,n,C,D^	1.6 ± 0.4 ^p,o,n,B,C^	0.13 ± 0.04 ^p,B,C^	0.25 ± 0.08 ^p,o,C^
	6	21 ± 6 ^s,r,q,p,A,B^	5.4 ± 1.3 ^s,r,q,p,A,B^	0.45 ± 0.09 ^q,p,B^	1.2 ± 0.3 ^q,p,o,A,B^
	7	24 ± 5 ^u,t,s,A^	6.25 ± 1.13 ^t,s,A^	0.66 ± 0.18 ^r,q,A^	1.5 ± 0.3 ^r,q,A^
FP	4	1.07 ± 0.04 ^k,D^	0.28 ± 0.06 ^i,G^	0.054 ± 0.002 ^k,E^	0.145 ± 0.013 ^j,k,E^
	5	2.84 ± 1.07 ^j,k,D^	1.4 ± 0.7 ^g,h,F^	0.14 ± 0.08 ^i,j,k,E^	0.52 ± 0.18 ^h,i,D,E^
	6	10.3966 ± 1.0114 ^h,i,j,C^	3.0 ± 0.4 ^d,e,D,E^	0.66 ± 0.04 ^f,g,D^	1.8 ± 0.2 ^e,C^
	7	19.5 ± 1.7 ^g,B^	4.6 ± 0.4 ^c,C^	1.11 ± 0.13 ^e,C^	3.0 ± 0.3 ^d,B^
65FP	4	3.90 ± 1.08 ^p,o,n,D^	2.2 ± 0.5 ^q,p,o,E,F^	0.29 ± 0.18 ^q,p,E^	0.73 ± 0.08 ^q,p,o,D^
	5	11.1 ± 1.4 ^s,r,q,p,C^	3.9 ± 0.3 ^t,s,r,q,C,D^	0.76 ± 0.15 ^s,r,D^	1.9 ± 0.4 ^t,s,C^
	6	22 ± 5 ^u,t,B^	5.5 ± 0.9 ^u,t,B^	1.5 ± 0.3 ^u,A^	3.2 ± 0.5 ^u,B^
	7	41 ± 6 ^v,A^	9.7 ± 1.4 ^w,A^	2.7 ± 0.6 ^w,B^	5.5 ± 0.8 ^v,A^
FC	4	1.004 ± 0.013 ^k,C^	0.177 ± 0.008 ^i,B,C^	0.0720 ± 0.0014 ^j,k,E^	0.153 ± 0.008 ^j,k,D^
	5	1.058 ± 0.014 ^k,C^	0.1860 ± 0.0104 ^i,C^	0.100 ± 0.003 ^j,k,E^	0.170 ± 0.008 ^k,D^
	6	1.285 ± 0.009 ^k,B^	0.214 ± 0.006 ^i,B^	0.257 ± 0.004 ^h,i,j,k,C^	0.306 ± 0.013 ^i,j,k,C^
	7	1.68 ± 0.09 ^k,A^	0.26 ± 0.02 ^i,A^	0.37 ± 0.02 ^h,i,B^	0.47 ± 0.08 ^i,j,B^
65FC	4	0.970 ± 0.009 ^n,C^	0.178 ± 0.008 ^n,B,C^	0.0756 ± 0.0017 ^p,E^	0.152 ± 0.008 ^o,D^
	5	1.05 ± 0.03 ^n,C^	0.182 ± 0.002 ^n,B,C^	0.124 ± 0.009 ^p,D^	0.2050 ± 0.0113 ^p,o,D^
	6	1.28 ± 0.03 ^n,B^	0.209 ± 0.014 ^n,B^	0.246 ± 0.007 ^q,p,C^	0.33 ± 0.04 ^p,o,C^
	7	1.89 ± 0.06 ^o,n,A^	0.29580 ± 0.01006 ^n,A^	0.45 ± 0,03 ^r,q,p,A^	0.662 ± 0.119 ^q,p,o,A^
FBPC	4	6.5 ± 0.5 ^h,G^	1.267 ± 0.106 ^g,h,E^	0.71 ± 0.05 ^f,g,F^	1.05 ± 0.04 ^f,F^
	5	12.4 ± 1.5 ^f,g,F,G^	2.2 ± 0.3 ^e,f,g,E^	1.33 ± 0.07 ^d,e,E^	1.91 ± 0.10 ^e,E^
	6	27 ± 3 ^c,D,E^	4.7 ± 0.4 ^c,D^	2.26 ± 0.15 ^c,D^	3.22 ± 0.13 ^d,D^
	7	42 ± 8 ^a,C^	8 ± 2 ^a,C^	3.6 ± 0.5 ^b,C^	5.5 ± 0.7 ^b,C^
65FBPC	4	24 ± 2 ^u,E,F^	4.5 ± 0.6 ^t,s,r,D^	1.3 ± 0.2 ^u,t,E^	2.21 ± 0.16 ^t,E^
	5	39 ± 6 ^v,C,D^	7 ± 2 ^v,C^	2.20 ± 0.17 ^v,D^	4.0 ± 0.4 ^v,D^
	6	75 ± 13 ^x,B^	14 ± 2 ^y,B^	4.3 ± 0.5 ^x,B^	8.0 ± 0.9 ^y,B^
	7	107 ± 21 ^z,A^	20 ± 4 ^z,A^	6.0 ± 0.6 ^y,A^	10.4 ± 1.3 ^z,A^
FPESB	4	5.7 ± 0.7 ^h,i,E^	1.274 ± 0.103 ^g,h,E^	0.49 ± 0.06 ^g,h,E^	0.92 ± 0.19 ^f,g,E^
	5	14 ± 6 ^e,f,g,D^	2.5 ± 0.8 ^d,e,f,D,E^	1.3 ± 0.2 ^d,e,D^	2.1 ± 0.4 ^e,D^
	6	16.9 ± 0.7 ^c,D^	3.204 ± 0.115 ^d,D^	2.4 ± 0.2 ^c,C^	3.73 ± 0.14 ^c,C^
	7	36 ± 6 ^a,C^	6.6 ± 1.5 ^b,C^	4.2 ± 0.6 ^a,B^	6.2 ± 0.6 ^a,B^
65FPESB	4	18 ± 2 ^u,t,s,r,D^	3.7 ± 0.4 ^t,s,r,q,D^	1.24 ± 0.18 ^u,t,D^	1.8 ± 0.3 ^t,s,D^
	5	38 ± 5 ^v,C^	7.01 ± 1.04 ^v,u,C^	2.4 ± 0.3 ^w,v,C^	3.8 ± 0.4 ^v,C^
	6	63 ± 3 ^w,B^	12.08 ± 1.12 ^x,B^	4.5 ± 0.9 ^x,B^	6.4 ± 0.3 ^x,B^
	7	94 ± 9 ^y,A^	19 ± 2 ^z,A^	7.3 ± 0.4 ^z,A^	10.1 ± 0.4 ^z,A^

For the samples with the same conditions, the same lowercase letter in superscript in columns indicates the homogeneous groups established by ANOVA (*p* < 0.05) (a–n for unheated, z–k for heated samples). To compare the same sample with the temperature effect, the same capital superscript letter in columns indicates the homogeneous groups established by ANOVA (*p* < 0.05). C: Concentration (%).

**Table 6:** Mean values ± standard deviations of the rheology parameters *k* (consistency index), *n* (flow index) and *η*_ap_ (shear viscosity at 50 s^−1^ shear stress) of the formulated gels.

		Ostwald-De Waele Model			
Sample	C	*k* (Pa s*^n^*)	*n*	*η*_ap_ (Pa s)	*R* ^2^
FCH	4	1.74 ± 0.05 ^g,h,i,j,D,E^	0.519 ± 0.008 ^d,A^	0.265 ± 0.009 ^g,h,D^	0.99
	5	3.1 ± 0.8 ^f,g,C^	0.4991 ± 0.0103 ^d,e,B^	0.440530 ± 0.101015 ^e,f,C^	0.99
	6	3.61 ± 0.09 ^f,B,C^	0.502 ± 0.006 ^d,e,f,B^	0.515 ± 0.012 ^e,B^	0.99
	7	5.8 ± 0.6 ^e,A^	0.485 ± 0.005 ^e,f,g,h,C^	0.77 ± 0.06 ^d,A^	0.99
65FCH	4	1.55 ± 0.02 ^o,n,E^	0.519 ± 0.003 ^u,A^	0.2360 ± 0.0018 ^n,m,D^	0.99
	5	2.199 ± 0.116 ^p,o,D^	0.456 ± 0.006 ^t,s,r,q,D^	0.2618 ± 0.0104 ^o,n,m,D^	0.99
	6	3.93 ± 0.05 ^s,r,q,B^	0.507 ± 0.002 ^u,B^	0.570 ± 0.005 ^r,q,B^	0.99
	7	6.40 ± 0.04 ^u,t,A^	0.442 ± 0.002 ^r,q,E^	0.723 ± 0.008 ^t,s,A^	0.99
FPE	4	0.31 ± 0.06 ^j,k,C^	0.46 ± 0.04 ^i,B^	0.037 ± 0.009 ^k,C^	0.94
	5	0.28 ± 0.03 ^j,k,C^	0.5870 ± 0.0106 ^c,A^	0.056 ± 0.003 ^k,C^	0.96
	6	0.54 ± 0.17 ^i,j,k,B,C^	0.57 ± 0.05 ^c,A^	0.098 ± 0.017 ^j,k,B,C^	0.98
	7	6 ± 2 ^e,A^	0.264 ± 0.007 ^n,D^	0.35 ± 0.12 ^f,g,A^	0.95
65FPE	4	0.21 ± 0.03 ^n,C^	0.58 ± 0.03 ^x,A^	0.040 ± 0.003 ^k,C^	0.97
	5	0.55 ± 0.16 ^n,m,B,C^	0.56 ± 0.03 ^x,w,A^	0.098 ± 0.019 ^l,k,B,C^	0.98
	6	2.0 ± 0.6 ^p,o,B^	0.39 ± 0.05 ^p,C^	0.18 ± 0.03 ^m,l,B^	0.98
	7	5.16 ± 1.04 ^t,s,A^	0.3019 ± 0.0004 ^o,D^	0.34 ± 0.07 ^p,o,A^	0.97
FP	4	1.5 ± 0.3 ^h,i,j,k,D^	0.34 ± 0.03 ^k,l,A,B^	0.1117 ± 0.0106 ^i,j,k,D^	0.98
	5	2.8 ± 0.3 ^f,g,h,D^	0.34 ± 0.03 ^k,A^	0.216 ± 0.019 ^h,i,j,D^	0.97
	6	6.8 ± 0.9 ^e,C^	0.3115 ± 0.0115 ^l,m,A,B,C^	0.46 ± 0.04 ^e,f,C^	0.97
	7	13 ± 3 ^d,B^	0.30 ± 0.02 ^m,B,C,D^	0.81 ± 0.09 ^d,B^	0.98
65FP	4	3.052 ± 0.105 ^q,p,D^	0.277 ± 0.005 ^o,C,D^	0.180 ± 0.003 ^m,l,D^	0.99
	5	6.6 ± 0.3 ^u,C^	0.29 ± 0.02 ^o,n,C,D^	0.41 ± 0.04 ^p,C^	0.98
	6	12.3 ± 1.5 ^w,v,B^	0.302 ± 0.013 ^o,B,C,D^	0.80 ± 0.07 ^t,B^	0.98
	7	22 ± 2 ^x,A^	0.275 ± 0.005 ^n,D^	1.27 ± 0.13 ^u,A^	0.98
FC	4	0.1017 ± 0.0009 ^k,F^	0.7151 ± 0.0014 ^a,A^	0.03338 ± 0.00018 ^k,F^	0.99
	5	0.338 ± 0.009 ^j,k,E,F^	0.651 ± 0.002 ^b,B^	0.086 ± 0.002 ^j,k,E^	0.99
	6	0.99 ± 0.04 ^i,j,k,D^	0.602 ± 0.006 ^c,C^	0.209 ± 0.005 ^h,i,j,D^	0.99
	7	2.631 ± 0.019 ^f,g,h,B^	0.578 ± 0.013 ^c,D^	0.51 ± 0.03 ^e,B^	0.99
65FC	4	0.130 ± 0.005 ^m,F^	0.716 ± 0.005 ^z,A^	0.0427 ± 0.0008 ^k,F^	0.99
	5	0.48 ± 0.03 ^n,m,E^	0.642 ± 0.008 ^y,B^	0.118 ± 0.003 ^l,k,E^	0.99
	6	1.51 ± 0.06 ^o,n,C^	0.585 ± 0.005 ^x,D^	0.297 ± 0.006 ^o,n,C^	0.99
	7	3.6 ± 0.4 ^r,q,A^	0.55393 ± 0.01019 ^w,E^	0.63 ± 0.04 ^r,q,A^	0.99
FBPC	4	1.98 ± 0.04 ^g,h,i,F^	0.458 ± 0.013 ^h,i,A^	0.2385 ± 0.0115 ^g,h,i,F^	0.99
	5	5.9 ± 0.6 ^e,E^	0.4682 ± 0.0015 ^g,h,i,A^	0.74 ± 0.08 ^d,E^	0.99
	6	17.2 ± 1.6 ^c,C^	0.462 ± 0.002 ^h,i,A^	2.10 ± 0.18 ^b,C^	0.99
	7	26.5 ± 1.5 ^a,A^	0.424 ± 0.009 ^j,B^	2.8 ± 0.2 ^a,A^	0.99
65FBPC	4	5.2 ± 0.2 ^t,s,E^	0.467 ± 0.013 ^t,s,A^	0.64 ± 0.06 ^s,r,E^	0.99
	5	12.0 ± 0.8 ^v,D^	0.46360 ± 0.01013 ^t,s,r,A^	1.47 ± 0.06 ^v,D^	0.99
	6	22.9 ± 1.9 ^y,B^	0.432 ± 0.005 ^q,B^	2.48 ± 0.17 ^x,B^	0.99
FPESB	4	2.09 ± 0.09 ^f,g,h,i,F^	0.496 ± 0.004 ^d,e,f,g,A^	0.290 ± 0.009 ^g,h,G^	0.99
	5	5.4 ± 0.4 ^e,E^	0.487 ± 0.006 ^e,f,g,h,A,B^	0.72 ± 0.04 ^d,E^	0.99
	6	11.1 ± 1.3 ^d,D^	0.473 ± 0.014 ^f,g,h,i,C,D^	1.41 ± 0.09 ^c,D^	0.99
	7	22.87 ± 1.13 ^b,B^	0.462 ± 0.006 ^h,i,D,E,F^	2.78 ± 0.09 ^a,B^	0.99
65FPESB	4	4.68 ± 0.16 ^s,r,E^	0.450 ± 0.005 ^s,r,q,F^	0.54384 ± 0.01009 ^q,F^	0.99
	5	13.5 ± 0.3 ^w,C^	0.459 ± 0.006 ^t,s,r,E,F^	1.629 ± 0.016 ^w,C^	0.99
	6	21.8 ± 0.5 ^y,x,B^	0.476 ± 0.003 ^t,s,r,B,C^	2.81 ± 0.06 ^y,B^	0.99
	7	36.7 ± 0.7 ^z,A^	0.466 ± 0.003 ^t,C,D,E^	4.55 ± 0.04 ^z,A^	0.99

For the samples with the same conditions, the same lowercase letter in superscript in columns indicates the homogeneous groups established by ANOVA (*p* < 0.05) (a–k for unheated, z–k for heated samples). To compare the same sample with the temperature effect, the same capital superscript letter in columns indicates the homogeneous groups established by ANOVA (*p* < 0.05). C: Concentration (%).

## Data Availability

The data herein presented are available on request from the corresponding author.
